# Hyperuricemia in hospitalized patients with heart failure: prevalence and clinical correlates

**DOI:** 10.3389/fmed.2026.1848140

**Published:** 2026-07-15

**Authors:** Lingqin Li, Xinzhu Yuan, Yanni Zhang, Quanbo Zhang, Yufeng Qing

**Affiliations:** 1 Department of Rheumatology and Immunology, Affiliated Hospital of North Sichuan Medical College, Nanchong, China; 2 Department of Nephrology, Beijing Anzhen Nanchong Hospital of Capital Medical University, Nanchong Central Hospital, Nanchong, China; 3 Department of Nephrology, Affiliated Hospital of North Sichuan Medical College, Nanchong, China; 4 Department of Geriatrics, Affiliated Hospital of North Sichuan Medical College, Nanchong, China

**Keywords:** clinical correlates, heart failure, hyperuricemia, prevalence, renal function

## Abstract

**Background:**

Hyperuricemia frequently accompanies heart failure (HF), yet hospital-based real-world data from China remain insufficient. We therefore evaluated the burden of hyperuricemia in hospitalized patients with HF and explored its associated clinical correlates.

**Methods:**

We carried out a retrospective cross-sectional analysis of 1,985 patients admitted with HF. Hyperuricemia was defined as serum uric acid ≥ 7.0 mg/dL in males and ≥ 6.0 mg/dL in females. Candidate correlates were examined using univariable and multivariable logistic regression models. To further characterize the findings, we additionally applied restricted cubic spline analyses, and subgroup analyses by sex and age, and NYHA class.

**Results:**

The overall prevalence of hyperuricemia was 69.42%. Prevalence was similar across sex, age, and BMI groups, but differed by NYHA class. In the multivariable model, hyperuricemia was independently associated with markers of renal dysfunction, hemodynamic impairment, electrolyte imbalance, and metabolic disturbance. RCS analyses suggested nonlinear associations for potassium, BNP, eGFR, and HDL-C.

**Conclusion:**

Hyperuricemia was highly prevalent among hospitalized patients with HF and was associated with multiple markers of renal dysfunction, hemodynamic impairment, electrolyte imbalance, and metabolic disturbance. These findings provide real-world evidence suggesting that hyperuricemia in hospitalized HF patients may reflect a broader cardiorenal and metabolic profile.

## 1 Introduction

Hyperuricemia refers to an abnormal elevation in serum uric acid and is often accompanied by reduced urate excretion (1). Over recent years, its prevalence has risen globally, with estimates ranging from 2.6 to 36% in different populations (2, 3). In China, more than 15.4% of adults are affected, and the condition is more frequent in males than in females (4). In addition to serving as the biochemical basis of gout, hyperuricemia has been associated with a range of chronic disorders, including hypertension, chronic kidney disease, diabetes mellitus, and metabolic syndrome (5). Elevated serum uric acid has also been associated with inflammation, endothelial injury, oxidative imbalance, and a higher burden of cardiovascular events (6).

Heart failure (HF) is a clinically complex syndrome with poor prognosis and is often accompanied by metabolic abnormalities (7). Among these, hyperuricemia is particularly common, with some studies reporting prevalence as high as 43% (8). This high burden may be related, at least in part, to reduced renal perfusion and the widespread use of diuretics in patients with HF (9). Previous studies have also shown that hyperuricemia in HF is associated with adverse cardiac remodeling, lower left ventricular ejection fraction, and a greater burden of hospitalization and mortality (10–13).

Despite the frequent coexistence of hyperuricemia and HF, large real-world hospital-based evidence from China remains limited. In addition, the epidemiological characteristics of hyperuricemia in hospitalized HF patients, as well as its clinical correlates across sex, age, and HF severity strata, have not been fully characterized. Therefore, this study used a hospital-based HF database derived from the electronic medical records of a tertiary care center in China to estimate the prevalence of hyperuricemia, identify its clinical correlates, and examine the consistency of these associations across clinically relevant subgroups.

## 2 Methods

### 2.1 Data source

This study was based on an inpatient HF dataset developed at the Fourth People’s Hospital of Zigong, Sichuan, China. The database included all first recorded hospital admissions for HF from December 2016 to June 2019. It was a single-center retrospective dataset containing structured clinical information derived from the hospital’s electronic medical record system. Zhang et al. (14) later made a de-identified version of this dataset publicly accessible through PhysioNet.

### 2.2 Study design and population

This investigation was conducted as a retrospective cross-sectional study using the inpatient HF database. The diagnosis of HF followed the 2016 European Society of Cardiology guidelines (15), which required typical symptoms and signs together with objective evidence of cardiac structural or functional abnormalities, including elevated left ventricular filling pressures, and/or increased natriuretic peptide levels. When the diagnosis was uncertain, additional transthoracic echocardiography, natriuretic peptide testing, or invasive hemodynamic evaluation was performed according to clinical need. Patients with missing key clinical information or incomplete medical records were excluded.

### 2.3 Definition of hyperuricemia

Hyperuricemia was classified according to serum uric acid concentration. Patients were considered to have hyperuricemia when serum uric acid was ≥ 7.0 mg/dL in males or ≥ 6.0 mg/dL in females (16). Blood samples were obtained in the early morning at admission and processed in the hospital clinical laboratory with a standardized automated assay system.

### 2.4 Variables and covariates

Variables retrieved from the inpatient HF database covered demographic, clinical, laboratory, and treatment-related domains. Demographic variables included sex and age. Age was categorized as <70 years and ≥70 years for subgroup analyses. Clinical variables included HF type, New York Heart Association (NYHA) class, body mass index (BMI), systolic blood pressure (SBP), and diastolic blood pressure (DBP). Laboratory measures comprised white blood cell count (WBC), monocyte count, red blood cell count (RBC), hemoglobin, platelet count, serum creatinine (SCr), estimated glomerular filtration rate (eGFR), calcium, potassium, B-type natriuretic peptide (BNP), albumin, globulin, high-sensitivity cardiac troponin (hs-cTn), triglyceride, low-density lipoprotein cholesterol (LDL-C), and high-density lipoprotein cholesterol (HDL-C). Medication-related variables included statin use, angiotensin-converting enzyme inhibitors or angiotensin receptor blockers (ACEI/ARB), diuretics, and beta-blockers. Medication exposure was recorded as binary use or non-use variables.

### 2.5 Statistical analysis

R software (version 4.3.3) and DecisionLinnc (version 1.0) were used for all analyses (17). Continuous data are presented as mean ± standard deviation when distributional assumptions were approximately met, and as median with interquartile range otherwise. Categorical data are reported as counts with percentages. Group comparisons between patients with and without hyperuricemia were carried out using the t-test or Mann–Whitney U test for continuous variables and the chi-square test for categorical variables. We also calculated the overall prevalence of hyperuricemia and summarized prevalence across predefined subgroups.

To examine factors associated with hyperuricemia, logistic regression models were fitted with hyperuricemia status as the dependent variable. Univariable logistic regression analyses were first conducted for each candidate variable, and odds ratios (ORs) with 95% CIs were estimated. Variables showing a *p* value < 0.05 in univariable analyses, together with variables considered clinically relevant, were subsequently included in the multivariable logistic regression model to obtain adjusted ORs and 95% CIs. Multicollinearity among candidate covariates was assessed using variance inflation factors, and highly collinear variables were not entered simultaneously into the same multivariable model. To further evaluate potential heterogeneity, multivariable logistic regression analyses were additionally repeated in sex-, age-, and NYHA-stratified subgroups, with interaction testing performed for each stratification variable.

For continuous variables that remained significantly associated with hyperuricemia in the multivariable model, restricted cubic spline (RCS) analyses were further performed to explore the shape of their associations with hyperuricemia. Four knots were specified at the 5th, 35th, 65th, and 95th percentiles, and the second knot was used as the reference. All analyses were two-tailed, and statistical significance was defined as *p* < 0.05.

## 3 Results

### 3.1 Baseline characteristics and prevalence of hyperuricemia

**Figure 1:**
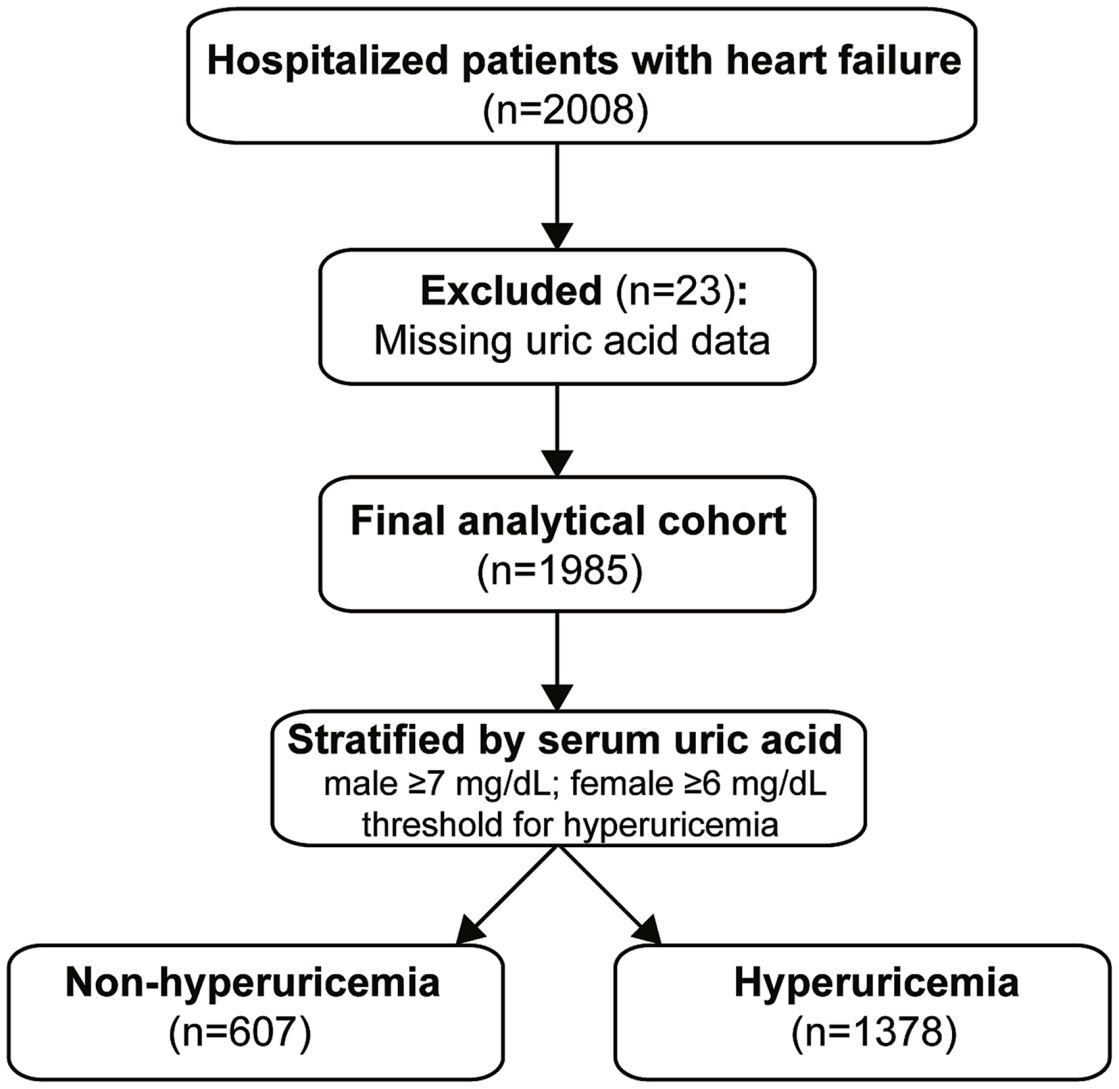
Flow chart of the study population.

A total of 1,985 hospitalized patients with HF were included in the final analysis (Figure 1). Among them, 1,378 patients had hyperuricemia, with an overall prevalence of 69.42%. The prevalence of hyperuricemia was comparable between males and females (68.78% vs. 69.89%) and between patients aged <70 years and those aged ≥70 years (72.17% vs. 68.40%). Baseline characteristics according to hyperuricemia status are shown in Table 1. Compared with patients without hyperuricemia, those with hyperuricemia had more advanced NYHA class, lower SBP and DBP, worse renal function, higher BNP, higher inflammatory and electrolyte-related markers, and lower HDL-C. By contrast, the two groups were broadly comparable in terms of sex, age group, heart failure type, BMI, RBC count, hemoglobin, platelet count, albumin, LDL-C, ACEI/ARB use, diuretic use, and beta-blocker use.

**Table 1:** Baseline characteristics of study population.

Variable	Non-hyperuricemia (*n* = 607)	Hyperuricemia (*n* = 1,378)	*p*-value
Sex, *n* (%)			
Female	346 (57.00%)	803 (58.27%)	0.632
Male	261 (43.00%)	575 (41.73%)	
Age, *n* (%)			
<70	150 (24.71%)	389 (28.23%)	0.117
≥70	457 (75.29%)	989 (71.77%)	
Heart failure type, *n* (%)			
Left	153 (25.21%)	315 (22.86%)	0.487
Right	14 (2.31%)	37 (2.69%)	
Both	440 (72.49%)	1,026 (74.46%)	
NYHA class, *n* (%)			
II	116 (19.11%)	231 (16.76%)	0.014
III	332 (54.70%)	696 (50.51%)	
IV	159 (26.19%)	451 (32.73%)	
BMI, kg/m^2^	20.76 (18.37, 23.44)	20.76 (18.54, 23.44)	0.833
SBP, mmHg	136.02 ± 24.81	128.77 ± 24.27	<0.001
DBP, mmHg	79.41 ± 13.91	75.27 ± 14.55	<0.001
WBC, ×10^9^/L	6.12 (4.83, 8.00)	6.65 (5.21, 8.89)	<0.001
Monocyte, ×10^9^/L	0.39 (0.30, 0.51)	0.44 (0.33, 0.61)	<0.001
RBC, ×10^12^/L	3.90 ± 0.68	3.84 ± 0.80	0.097
Hemoglobin, g/L	116.41 ± 22.57	114.52 ± 25.37	0.101
Platelet, ×109/L	134.00 (101.00, 175.50)	135.00 (101.00, 177.00)	0.927
SCr, μmol/L	65.70 (54.35, 84.10)	99.25 (74.53, 137.18)	<0.001
eGFR, mL/min/1.73m^2^	89.45 (67.49, 109.51)	54.50 (36.02, 75.91)	<0.001
Calcium, mmol/L	2.27 ± 0.18	2.30 ± 0.18	<0.001
Potassium, mmol/L	3.75 (3.44, 4.05)	3.95 (3.58, 4.43)	<0.001
BNP, pg./mL	526.05 (222.19, 1143.61)	916.67 (372.87, 1958.81)	<0.001
Albumin, g/L	36.41 ± 4.68	36.59 ± 5.10	0.475
Globulin, g/L	27.50 (24.40, 30.90)	28.20 (24.80, 31.60)	0.016
hs-cTn, ng/mL	0.03 (0.01, 0.08)	0.06 (0.03, 0.13)	<0.001
Triglyceride, mmol/L	0.90 (0.68, 1.18)	0.99 (0.73, 1.36)	<0.001
LDL-C, mmol/L	1.77 (1.36, 2.25)	1.75 (1.30, 2.30)	0.734
HDL-C, mmol/L	1.17 (0.94, 1.38)	1.04 (0.82, 1.26)	<0.001
Statin, *n* (%)			
No	337 (55.52%)	832 (60.38%)	0.046
Yes	270 (44.48%)	545 (39.55%)	
ACEI/ARB, *n* (%)			
No	357 (58.81%)	864 (62.70%)	0.108
Yes	250 (41.19%)	513 (37.23%)	
Diuretic, *n* (%)			
No	14 (2.31%)	17 (1.23%)	0.115
Yes	593 (97.69%)	1,360 (98.69%)	
Beta-blocker, *n* (%)			
No	384 (63.26%)	843 (61.18%)	0.416
Yes	223 (36.74%)	534 (38.75%)	

NYHA, New York Heart Association; BMI, body mass index; SBP, systolic blood pressure; DBP, diastolic blood pressure; WBC, white blood cell; RBC, red blood cell; SCr, serum creatinine; eGFR, estimated glomerular filtration rate; BNP, B-type natriuretic peptide; hs-cTn, high-sensitivity cardiac troponin; LDL-C, low-density lipoprotein cholesterol; HDL-C, high-density lipoprotein cholesterol; ACEI/ARB, angiotensin-converting enzyme inhibitor/angiotensin receptor blocker.

### 3.2 Logistic regression analyses of factors associated with hyperuricemia

**Figure 2:**
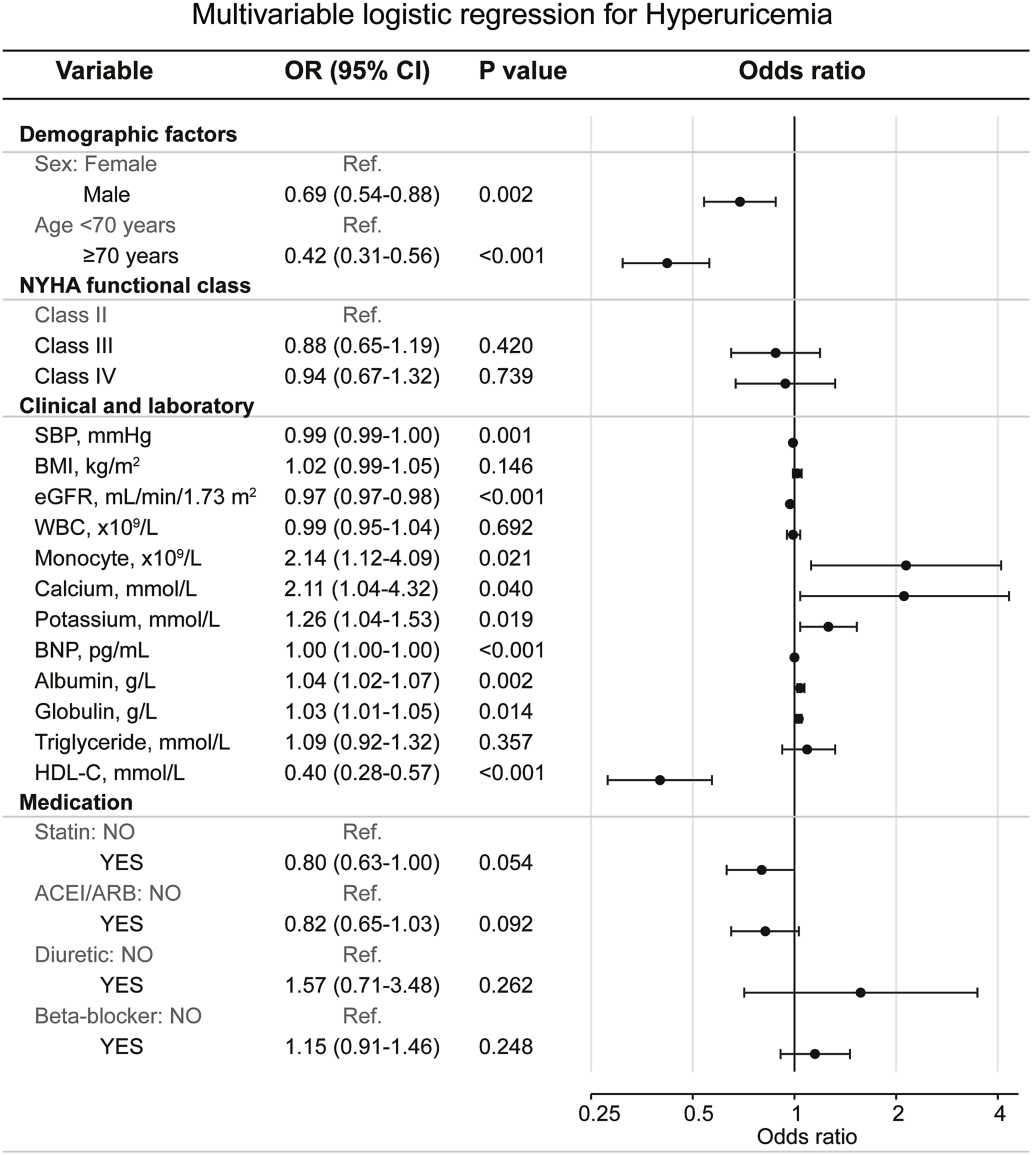
Multivariable logistic regression analysis. NYHA, New York Heart Association; SBP, systolic blood pressure; BMI, body mass index; eGFR, estimated glomerular filtration rate; WBC, white blood cell; BNP, B-type natriuretic peptide; HDL-C, high-density lipoprotein cholesterol; ACEI/ARB, angiotensin-converting enzyme inhibitor/angiotensin receptor blocker.

In the univariable analyses, NYHA class IV, lower blood pressure, impaired renal function, higher BNP, higher potassium, and lower HDL-C were significantly associated with hyperuricemia (Table 2). The multivariable model was constructed based on the univariable findings and clinical relevance (Figure 2). Male sex (OR = 0.69, *p* = 0.002) and age ≥70 years (OR = 0.42, *p* < 0.001) were associated with lower odds of hyperuricemia. Lower SBP (OR = 0.99, *p* = 0.001), lower eGFR (OR = 0.97, *p* < 0.001), and lower HDL-C (OR = 0.40, *p* < 0.001) were associated with higher odds of hyperuricemia. Higher monocyte count, calcium, potassium, BNP, albumin, and globulin were also positively associated with hyperuricemia. The VIF results are provided in Supplementary Table S1, and no substantial multicollinearity was observed.

**Table 2:** Univariate logistic regression.

Variable	OR	95% CI	*p* value
Sex, *n* (%)			
Female	1.00		
Male	0.95	0.78–1.15	0.597
Age, *n* (%)			
<70	1.00		
≥70	0.83	0.67–1.04	0.105
Heart failure type, *n* (%)			
Left	1.00		
Right	1.28	0.69–2.52	0.448
Both	1.13	0.90–1.41	0.274
NYHA class, *n* (%)			
II	1.00		
III	1.05	0.81–1.36	0.697
IV	1.42	1.07–1.90	0.016
BMI, kg/m^2^	1.01	0.98–1.03	0.607
SBP, mmHg	0.99	0.98–0.99	<0.001
DBP, mmHg	0.98	0.97–0.99	<0.001
WBC, ×10^9^/L	1.06	1.02–1.09	<0.001
Monocyte, ×10^9^/L	2.84	1.84–4.47	<0.001
RBC, ×10^12^/L	0.91	0.80–1.03	0.120
Hemoglobin, g/L	1.00	0.99–1.00	0.119
Platelet, ×109/L	1.00	1.00–1.00	0.644
SCr, μmol/L	1.02	1.01–1.02	<0.001
eGFR, mL/min/1.73 m^2^	0.98	0.97–0.98	<0.001
Calcium, mmol/L	2.71	1.59–4.66	<0.001
Potassium, mmol/L	1.89	1.62–2.23	<0.001
BNP, pg./mL	1.00	1.00–1.00	<0.001
Albumin, g/L	1.01	0.99–1.03	0.484
Globulin, g/L	1.03	1.01–1.04	0.004
hs-cTn, ng/mL	1.14	0.97–1.34	0.105
Triglyceride, mmol/L	1.33	1.14–1.58	0.001
LDL-C, mmol/L	0.99	0.87–1.12	0.845
HDL-C, mmol/L	0.38	0.28–0.50	<0.001
Statin, *n* (%)			
No	1.00		
Yes	0.82	0.68–0.99	0.043
ACEI/ARB, *n* (%)			
No	1.00		
Yes	0.85	0.70–1.03	0.095
Diuretic, *n* (%)			
No	1.00		
Yes	1.89	0.91–3.86	0.080
Beta-blocker, *n* (%)			
No	1.00		
Yes	1.09	0.90–1.33	0.378

NYHA, New York Heart Association; BMI, body mass index; SBP, systolic blood pressure; DBP, diastolic blood pressure; WBC, white blood cell; RBC, red blood cell; SCr, serum creatinine; eGFR, estimated glomerular filtration rate; BNP, B-type natriuretic peptide; hs-cTn, high-sensitivity cardiac troponin; LDL-C, low-density lipoprotein cholesterol; HDL-C, high-density lipoprotein cholesterol; ACEI/ARB, angiotensin-converting enzyme inhibitor/angiotensin receptor blocker.

To further assess the influence of sex-specific diagnostic thresholds, a sensitivity analysis was performed using a common hyperuricemia threshold of serum uric acid ≥7.0 mg/dL for both males and females. In this analysis, male sex was associated with higher odds of hyperuricemia (OR = 1.82, *p* < 0.001), whereas age ≥70 years remained associated with lower odds of hyperuricemia (OR = 0.45, *p* < 0.001). Lower SBP, lower eGFR, higher monocyte count, higher calcium, higher potassium, higher BNP, higher albumin, and lower HDL-C remained significantly associated with hyperuricemia (Supplementary Table S2).

To explore whether HF severity modified the associations of sex and age with hyperuricemia, exploratory NYHA-stratified analyses were performed. Patients were stratified into NYHA II/III and NYHA IV groups. In these analyses, male sex was associated with lower odds of hyperuricemia in both NYHA strata, although the association was statistically significant only in the NYHA IV stratum. Age ≥70 years was consistently associated with lower odds of hyperuricemia in both NYHA strata. No significant interaction was observed between sex and NYHA class or between age group and NYHA class (Supplementary Table S3).

### 3.3 Sex and age stratified analyses

Sex- and age-stratified analyses are presented in Tables 3, 4. In the sex-stratified analyses, age ≥70 years was associated with lower odds of hyperuricemia in both females and males, with no significant interaction by sex. Lower eGFR, higher BNP, and lower HDL-C were consistently associated with hyperuricemia in both sex strata, whereas significant interactions by sex were observed for eGFR and diuretic use. In the age-stratified analyses, male sex was associated with lower odds of hyperuricemia in both patients aged <70 years and those aged ≥70 years, with no significant interaction by age group. Lower eGFR, higher BNP, higher albumin, and lower HDL-C were consistently associated with hyperuricemia in both age strata, whereas significant interactions by age group were observed for eGFR and globulin.

**Table 3:** Sex stratified correlates of hyperuricemia in HF patients.

Variable	Female			Male			P interaction
	OR	95% CI	*p*-value	OR	95% CI	*p*-value	
Age, *n* (%)							0.755
<70	1.00			1.00			
≥70	0.34	0.22–0.52	<0.001	0.58	0.38–0.89	0.013	
NYHA class, *n* (%)							0.273
II	1.00			1.00			
III	0.80	0.54–1.18	0.263	0.98	0.60–1.58	0.932	
IV	1.01	0.64–1.60	0.961	0.88	0.52–1.48	0.643	
SBP, mmHg	0.99	0.99–1.00	0.017	0.99	0.98–1.00	0.033	0.847
BMI, kg/m^2^	1.02	0.98–1.06	0.421	1.03	0.99–1.08	0.157	0.408
eGFR, mL/min/1.73 m^2^	0.97	0.96–0.97	<0.001	0.98	0.97–0.98	<0.001	0.013
WBC, ×10^9^/L	0.96	0.90–1.02	0.190	1.04	0.97–1.12	0.294	0.588
Monocyte, ×10^9^/L	3.65	1.48–9.15	0.005	1.20	0.45–3.14	0.716	0.386
Calcium, mmol/L	2.87	1.12–7.50	0.029	1.47	0.48–4.54	0.499	0.315
Potassium, mmol/L	1.25	0.96–1.64	0.101	1.28	0.96–1.72	0.098	0.471
BNP, pg./mL	1.00	1.00–1.00	<0.001	1.00	1.00–1.00	0.003	0.178
Albumin, g/L	1.06	1.02–1.10	0.004	1.03	0.99–1.08	0.127	0.660
Globulin, g/L	1.04	1.01–1.07	0.007	1.01	0.98–1.04	0.677	0.176
Triglyceride, mmol/L	1.05	0.88–1.33	0.639	1.21	0.85–1.76	0.311	0.343
HDL-C, mmol/L	0.43	0.27–0.71	0.001	0.34	0.19–0.59	<0.001	0.304
Statin, *n* (%)							0.883
No	1.00			1.00			
Yes	0.81	0.59–1.11	0.198	0.73	0.52–1.03	0.077	
ACEI/ARB, *n* (%)							0.967
No	1.00			1.00			
Yes	0.81	0.58–1.12	0.204	0.79	0.56–1.13	0.195	
Diuretic, *n* (%)							0.020
No	1.00			1.00			
Yes	3.58	1.23–11.16	0.022	0.52	0.11–1.91	0.358	
Beta-blocker, *n* (%)							0.307
No	1.00			1.00			
Yes	1.06	0.77–1.47	0.723	1.30	0.91–1.88	0.150	

NYHA, New York Heart Association; SBP, systolic blood pressure; BMI, body mass index; eGFR, estimated glomerular filtration rate; WBC, white blood cell; BNP, B-type natriuretic peptide; HDL-C, high-density lipoprotein cholesterol; ACEI/ARB, angiotensin-converting enzyme inhibitor/angiotensin receptor blocker.

**Table 4:** Age-stratified correlates of hyperuricemia in HF patients.

Variable	<70			≥70			*p* interaction
	OR	95% CI	*p*-value	OR	95% CI	*p*-value	
Sex, *n* (%)							0.755
Female	1.00			1.00			
Male	0.61	0.38–0.97	0.037	0.69	0.52–0.91	0.009	
NYHA class, *n* (%)							0.906
II	1.00			1.00			
III	0.98	0.55–1.72	0.940	0.86	0.60–1.23	0.407	
IV	1.13	0.58–2.17	0.718	0.92	0.61–1.37	0.671	
SBP, mmHg	0.99	0.98–1.00	0.105	0.99	0.99–1.00	0.004	0.977
BMI, kg/m^2^	1.00	0.95–1.06	0.937	1.02	0.99–1.06	0.184	0.769
eGFR, mL/min/1.73 m^2^	0.98	0.97–0.98	<0.001	0.97	0.96–0.97	<0.001	0.037
WBC, ×10^9^/L	1.01	0.92–1.10	0.889	0.99	0.93–1.04	0.589	0.588
Monocyte, ×10^9^/L	2.66	0.80–9.26	0.117	2.12	0.99–4.54	0.053	0.640
Calcium, mmol/L	2.30	0.62–8.83	0.218	2.18	0.91–5.26	0.080	0.720
Potassium, mmol/L	0.97	0.67–1.44	0.877	1.35	1.08–1.70	0.009	0.066
BNP, pg./mL	1.00	1.00–1.00	0.007	1.00	1.00–1.00	<0.001	0.682
Albumin, g/L	1.05	1.00–1.11	0.034	1.05	1.01–1.08	0.011	0.766
Globulin, g/L	0.96	0.91–1.00	0.038	1.05	1.02–1.07	<0.001	0.001
Triglyceride, mmol/L	1.19	0.93–1.64	0.248	0.99	0.81–1.28	0.918	0.375
HDL-C, mmol/L	0.34	0.16–0.72	0.005	0.40	0.26–0.61	<0.001	0.895
Statin, *n* (%)							0.874
No	1.00			1.00			
Yes	0.77	0.48–1.24	0.281	0.82	0.63–1.07	0.146	
ACEI/ARB, *n* (%)							0.930
No	1.00			1.00			
Yes	0.88	0.55–1.40	0.595	0.82	0.62–1.09	0.170	
Diuretic, *n* (%)							0.775
No	1.00			1.00			
Yes	2.53	0.38–16.40	0.315	1.51	0.61–3.70	0.363	
Beta-blocker, *n* (%)							0.139
No	1.00			1.00			
Yes	0.76	0.47–1.21	0.241	1.36	1.02–1.81	0.035	

NYHA, New York Heart Association; SBP, systolic blood pressure; BMI, body mass index; eGFR, estimated glomerular filtration rate; WBC, white blood cell; BNP, B-type natriuretic peptide; HDL-C, high-density lipoprotein cholesterol; ACEI/ARB, angiotensin-converting enzyme inhibitor/angiotensin receptor blocker.

### 3.4 Restricted cubic spline analyses

**Figure 3:**
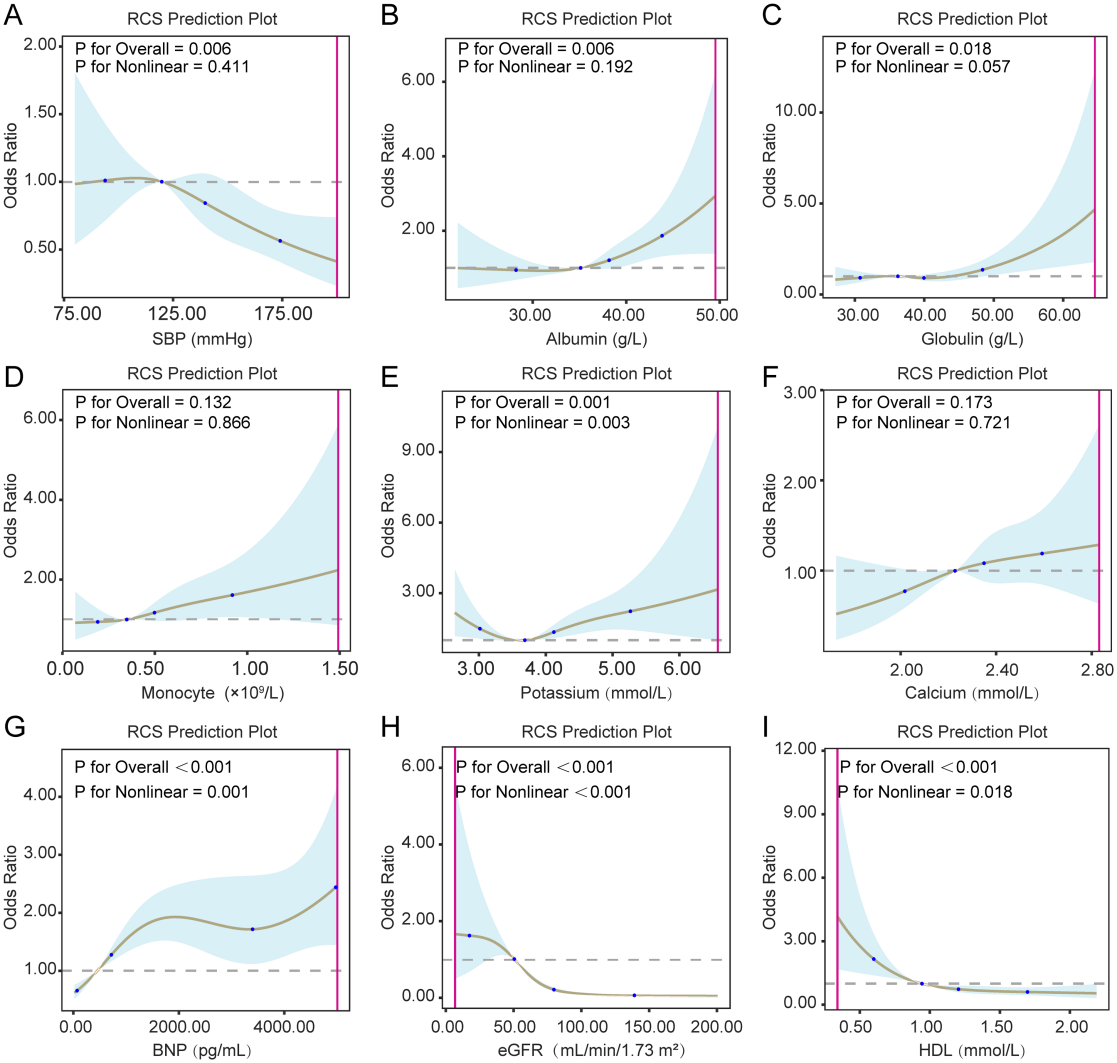
Nonlinear associations of continuous variables with hyperuricemia. **(A)** SBP, **(B)** albumin, **(C)** globulin, **(D)** monocyte, **(E)** potassium, **(F)** calcium, **(G)** BNP, **(H)** eGFR, **(I)** HDL-C. RCS, restricted cubic spline; SBP, systolic blood pressure; BNP, B-type natriuretic peptide; eGFR, estimated glomerular filtration rate; HDL-C, high-density lipoprotein cholesterol.

Restricted cubic spline analyses were further performed for continuous variables that remained significantly associated with hyperuricemia in the multivariable model (Figure 3). Significant overall associations were observed for SBP, albumin, globulin, potassium, BNP, eGFR, and HDL-C (all P for overall < 0.05). Among these, potassium, BNP, eGFR, and HDL-C also showed evidence of nonlinear associations (all P for nonlinearity < 0.05). Specifically, higher eGFR and HDL-C levels were associated with lower odds of hyperuricemia, whereas higher potassium and BNP levels were associated with higher odds of hyperuricemia in a nonlinear pattern. By contrast, the associations of SBP and albumin with hyperuricemia appeared approximately linear, with lower SBP and higher albumin being associated with higher odds of hyperuricemia. Globulin showed a borderline nonlinear association. No significant overall associations were observed for monocyte count or calcium in the spline models.

## 4 Discussion

In this hospital-based cross-sectional study of 1,985 patients with HF, hyperuricemia was highly prevalent, with an overall prevalence of 69.42%. In multivariable analyses, male sex and older age were associated with lower odds of hyperuricemia, whereas lower SBP, lower eGFR, lower HDL-C, and higher monocyte count, calcium, potassium, BNP, albumin, and globulin were independently associated with hyperuricemia. In addition, stratified analyses suggested that although several correlates were generally consistent across sex and age subgroups, some associations varied in magnitude and significance, indicating potential subgroup heterogeneity in the clinical profile of hyperuricemia among hospitalized patients with HF.

Hyperuricemia is common among patients with HF, but the reported prevalence varies considerably across cohorts. Previous studies reported prevalence rates of 59.96% in Japanese hospitalized HF patients (18), 59.52% in a Georgian HF cohort (19), and 57.6% in a Chinese HF cohort reported by Liu et al. (20). Lower estimates have also been reported, including 48.0% in central Ethiopia (21) and 40% in a Portuguese cohort (22). In our cohort, the prevalence of hyperuricemia was 69.42%, which was higher than most of these previous estimates. This relatively high prevalence may be related to the clinical profile of our study population. Our cohort consisted entirely of hospitalized HF patients and had an older age structure, suggesting a population with greater HF severity and cardiorenal burden than many ambulatory HF cohorts. In the baseline analysis, patients with hyperuricemia had more advanced NYHA class, lower SBP and DBP, and worse renal function, suggesting that hyperuricemia in this setting may be associated with hemodynamic stress and renal impairment. In addition, diuretic exposure was almost universal in our cohort, indicating substantial congestion or intensive volume management. These factors may have contributed to the higher prevalence of hyperuricemia observed in our study.

Among hospitalized patients with HF, the prevalence of hyperuricemia was similarly high in males and females, and no significant sex difference was observed in the descriptive analysis. A similar finding was reported by Liu in a cohort of hospitalized patients with HF (23). By contrast, Stubnova reported that, among ambulatory patients with chronic HF, males generally had higher serum uric acid levels than females (13). This discrepancy may partly reflect differences in study setting and patient composition. Compared with ambulatory populations, hospitalized patients with HF are usually older and have more advanced disease. In addition, many female patients in this setting may have been postmenopausal, in whom the uricosuric effect of estrogen is attenuated. These factors may reduce the usual sex difference in uric acid levels (24, 25).

However, the inverse association between male sex and hyperuricemia in the primary multivariable model required further evaluation. We found male sex (OR = 0.69) are associated with significantly lower odds of hyperuricemia. Recent literature has emphasized that conventional hyperuricemia thresholds may require recalibration in specific pathological contexts or high-risk populations (26). In addition, Zitt et al. (27) showed that the apparent sex difference in hyperuricemia prevalence changed markedly according to whether common or sex-specific cut-off points were used. Therefore, we performed a sensitivity analysis using a common serum uric acid threshold of ≥7.0 mg/dL for both males and females. Under this common-threshold definition, male sex was associated with higher odds of hyperuricemia, suggesting that the inverse association observed in the primary analysis was largely definition-dependent rather than evidence of a biological protective effect of male sex. This finding does not negate the primary sex-specific definition, but indicates that sex-related associations should be interpreted in light of the diagnostic threshold used.

The inverse association between age ≥70 years and hyperuricemia should be interpreted cautiously. Stubnova et al. (13) reported that patients with higher serum uric acid levels were older and had more severe HF symptoms, worse renal function, and greater diuretic use. Yan et al. (28) found that the hyperuricemia in elderly hospitalized HF patients was characterized by more advanced NYHA class, and more chronic kidney disease. In our study, the crude prevalence of hyperuricemia did not differ significantly by age group, and the inverse association for age ≥70 years appeared only after multivariable adjustment and persisted in the common-threshold sensitivity analysis. Therefore, this finding should be regarded as an adjusted conditional association within hospitalized HF patients rather than evidence that older age is biologically protective against hyperuricemia. Residual confounding from unmeasured comorbidities, frailty, nutritional status, urate-lowering therapy, and detailed diuretic exposure may also have contributed to this result (29).

Diuretic therapy is an important clinical factor in the relationship between HF and hyperuricemia. Loop and thiazide diuretics can increase serum uric acid levels by reducing renal urate excretion, promoting proximal tubular urate reabsorption, and inducing volume contraction (30). Therefore, the absence of a significant association between the binary variable of diuretic use and hyperuricemia in our study does not necessarily contradict the established biological effect of diuretics. In this hospitalized HF cohort, diuretic use was nearly universal: 97% of patients without hyperuricemia and 98% of those with hyperuricemia received diuretics. As a result, the non-use reference group was very small, and a simple yes/no variable provided limited contrast between groups. Thus, the true association between diuretic therapy and hyperuricemia may have been underestimated or obscured, particularly in the presence of confounding by congestion, HF severity, and renal function.

One of the most consistent findings in our study was that hyperuricemia was associated with markers of cardiorenal dysfunction and greater HF severity. Among the variables included in the multivariable model, eGFR showed the clearest inverse association with hyperuricemia, consistent with prior HF literature. Minneci et al. reported that the clinical significance of serum uric acid in HFrEF was closely conditioned by renal function (9), and earlier work also showed that changes in serum uric acid during acute HF treatment were related to renal function and diuretic dose (31). In our cohort, higher BNP and lower SBP were also associated with hyperuricemia, suggesting that hyperuricemia was more often observed in patients with a less favorable hemodynamic profile. This is in line with the study by Palazzuoli et al. (8), in which hyperuricemia was common in acute HF and was associated with worse clinical course. Potassium was also positively associated with hyperuricemia, particularly in older patients. Although this association has been less consistently reported, it may reflect the coexistence of renal impairment, neurohormonal activation, and treatment complexity in hospitalized HF. Overall, these findings suggest that hyperuricemia in HF may reflect a broader cardiorenal and hemodynamic disturbance.

In our study, higher serum calcium was associated with higher odds of hyperuricemia among hospitalized patients with HF. This finding is generally consistent with previous epidemiologic evidence. Liu et al. (32) reported a significant positive association between serum calcium and hyperuricemia, with individuals in the highest calcium quintile having approximately 2.5-fold higher odds of hyperuricemia than those in the lowest quintile. Analyses from NHANES also suggested a positive association between serum calcium and hyperuricemia, although the reported pattern was nonlinear (33). By contrast, in our study, the association identified in the multivariable model did not show a clearly nonlinear pattern in the spline analysis, suggesting that the shape of this association may vary across study populations. In patients with HF, disturbances in calcium homeostasis often coexist with neurohormonal activation, oxidative stress, and renal dysfunction, which may also overlap with abnormal uric acid metabolism (34–36). Therefore, serum calcium in this setting may reflect a broader metabolic and cardiorenal disturbance rather than an isolated biochemical change. Given the cross-sectional design, this association should be interpreted cautiously.

Higher HDL-C was independently associated with lower odds of hyperuricemia in our study, and this association remained generally consistent across the stratified and spline analyses. This finding suggests that HDL-C may be one component of the metabolic profile associated with hyperuricemia in hospitalized patients with HF. Previous studies have likewise shown that low HDL-C frequently coexists with hyperuricemia, particularly in individuals with metabolic syndrome and cardiovascular disease (37). However, the association between serum uric acid and lipid fractions may not be entirely uniform. A prospective study of healthy Japanese adults found that elevated serum uric acid was associated with hypertriglyceridemia and high LDL cholesterol, but not significantly associated with low HDL-C (38). These findings suggest that the relationship between uric acid and lipid metabolism may vary across populations and clinical settings. One possible explanation is that apolipoprotein A-I, the major structural protein of HDL, may also be reduced in hyperuricemia, potentially accompanying impaired HDL function and a less favorable oxidative and inflammatory profile (39–42). In this context, lower HDL-C in hospitalized patients with HF may represent a component of the broader metabolic disturbance associated with hyperuricemia, rather than an isolated lipid abnormality.

This real-world study provides a characterization of the epidemiology of hyperuricemia in patients with HF and its clinical correlates. Nevertheless, several limitations should be acknowledged. First, the single-centre retrospective cross-sectional design based on electronic health records may have introduced selection bias, information bias, and residual confounding; therefore, the observed associations should not be interpreted as causal or temporal relationships. Second, serum uric acid was assessed only at admission, and prognostic endpoints were unavailable, limiting our ability to evaluate longitudinal changes or the prognostic significance of hyperuricemia. Third, external validation in an independent cohort was not performed, which may limit the generalizability of the findings. Finally, several potential confounders, including lifestyle factors and detailed medication exposure, could not be fully captured; residual confounding therefore cannot be excluded (43). This constraint is common in real-world research and is difficult to fully avoid (44). In particular, information on diuretic class, dose, duration, route of administration, timing, and cumulative exposure was unavailable. Future multicentre prospective studies with longitudinal uric acid monitoring, detailed medication assessment, external validation, and prognostic follow-up are needed.

## 5 Conclusion

Hyperuricemia was highly prevalent in this cohort of hospitalized patients with HF. It was independently associated with multiple clinical factors, particularly indices of renal dysfunction, hemodynamic impairment, electrolyte imbalance, and metabolic disturbance. These findings provide real-world evidence suggesting that hyperuricemia in hospitalized HF patients may reflect a broader cardiorenal and metabolic profile rather than an isolated biochemical abnormality. Further multicentre prospective studies are needed to clarify its clinical relevance and prognostic significance in HF.

## Data Availability

Publicly available datasets were analyzed in this study. This data can be found at: https://doi.org/10.13026/8a9e-w734.
