# Supplementary material for: Hyperuricemia in hospitalized patients with heart failure: prevalence and clinical correlates
Source: Front Med (Lausanne). 2026 Jul 15;13:1848140. doi: 10.3389/fmed.2026.1848140 (PMC13416958; doi:10.3389/fmed.2026.1848140)
Supplement: Supplementary file 1 [file Table_1.docx]

| Supplementary Table S1. Multicollinearity for variables included in the multivariable logistic regression model | |
| --- | --- |
| Variable | GVIF^(1/(2*Df)) |
| Sex | 1.07 |
| Age | 1.17 |
| NYHA class | 1.02 |
| SBP, mmHg | 1.06 |
| BMI | 1.04 |
| eGFR | 1.17 |
| WBC | 1.31 |
| Monocyte | 1.31 |
| Calcium | 1.13 |
| Potassium | 1.04 |
| BNP | 1.07 |
| Albumin | 1.19 |
| Globulin | 1.03 |
| Triglyceride | 1.09 |
| HDL-C | 1.11 |
| Statin | 1.03 |
| ACEI/ARB | 1.05 |
| Diuretic | 1.02 |
| Beta-blocker | 1.06 |

Abbreviations: NYHA, New York Heart Association; SBP, systolic blood pressure; BMI, body mass index; eGFR, estimated glomerular filtration rate; WBC, white blood cell; BNP, B-type natriuretic peptide; HDL-C, high-density lipoprotein cholesterol; ACEI/ARB, angiotensin-converting enzyme inhibitor/angiotensin receptor blocker.
