# Supplementary material for: Hyperuricemia in hospitalized patients with heart failure: prevalence and clinical correlates
Source: Front Med (Lausanne). 2026 Jul 15;13:1848140. doi: 10.3389/fmed.2026.1848140 (PMC13416958; doi:10.3389/fmed.2026.1848140)
Supplement: Supplementary file 2 [file Table_2.docx]

| Supplementary Table S2. Sensitivity analysis using a common hyperuricemia threshold of serum uric acid ≥7.0 mg/dL in both males and females | | | |
| --- | --- | --- | --- |
| Variable | OR | 95% CI | P |
| Sex, n (%) |  |  |  |
| Female | 1.00 |  |  |
| Male | 1.82 | 1.45-2.28 | <0.001 |
| Age, n (%) |  |  |  |
| <70 | 1.00 |  |  |
| ≥70 | 0.45 | 0.34-0.60 | <0.001 |
| NYHA class, n (%) |  |  |  |
| II | 1.00 |  |  |
| III | 0.95 | 0.71-1.27 | 0.713 |
| IV | 1.01 | 0.73-1.40 | 0.945 |
| SBP, mmHg | 0.99 | 0.99-1.00 | <0.001 |
| BMI, kg/m^2^ | 1.01 | 0.98-1.04 | 0.406 |
| eGFR, mL/min/1.73 m^2^ | 0.97 | 0.97-0.98 | <0.001 |
| WBC, ×10⁹/L | 1.01 | 0.96-1.05 | 0.802 |
| Monocyte, ×10⁹/L | 2.12 | 1.16-3.90 | 0.015 |
| Calcium, mmol/L | 2.74 | 1.39-5.38 | 0.004 |
| Potassium, mmol/L | 1.20 | 1.00-1.43 | 0.047 |
| BNP, pg/mL | 1.00 | 1.00-1.00 | <0.001 |
| Albumin, g/L | 1.03 | 1.01-1.06 | 0.014 |
| Globulin, g/L | 1.01 | 0.99-1.03 | 0.148 |
| Triglyceride, mmol/L | 1.14 | 0.97-1.36 | 0.117 |
| HDL-C, mmol/L | 0.44 | 0.31-0.62 | <0.001 |
| Statin, n (%) |  |  |  |
| No | 1.00 |  |  |
| Yes | 0.75 | 0.60-0.93 | 0.010 |
| ACEI/ARB, n (%) |  |  |  |
| No | 1.00 |  |  |
| Yes | 0.87 | 0.69-1.09 | 0.209 |
| Diuretic, n (%) |  |  |  |
| No | 1.00 |  |  |
| Yes | 1.62 | 0.72-3.68 | 0.248 |
| Beta-blocker, n (%) |  |  |  |
| No | 1.00 |  |  |
| Yes | 1.10 | 0.87-1.38 | 0.431 |

Abbreviations: NYHA, New York Heart Association; SBP, systolic blood pressure; BMI, body mass index; eGFR, estimated glomerular filtration rate; WBC, white blood cell; BNP, B-type natriuretic peptide; HDL-C, high-density lipoprotein cholesterol; ACEI/ARB, angiotensin-converting enzyme inhibitor/angiotensin receptor blocker.
