# Supplementary material for: Hyperuricemia in hospitalized patients with heart failure: prevalence and clinical correlates
Source: Front Med (Lausanne). 2026 Jul 15;13:1848140. doi: 10.3389/fmed.2026.1848140 (PMC13416958; doi:10.3389/fmed.2026.1848140)
Supplement: Supplementary file 3 [file Table_3.docx]

| Supplementary Table S3. NYHA-Stratified multivariable correlates of Hyperuricemia in HF Patients | | | | | |
| --- | --- | --- | --- | --- | --- |
| Variable | NYHA II/III | | NYHA IV | | P interaction |
|  | OR (95% CI) | P | OR (95% CI) | P |  |
| Sex, n (%) |  |  |  |  | 0.127 |
| Female | 1.00 |  | 1.00 |  |  |
| Male | 0.75 (0.57-1.00) | 0.052 | 0.55 (0.35-0.86) | 0.008 |  |
| Age, n (%) |  |  |  |  | 0.958 |
| <70 | 1.00 |  | 1.00 |  |  |
| ≥70 | 0.39 (0.28-0.56) | <0.001 | 0.51 (0.29-0.89) | 0.017 |  |
| SBP, mmHg | 0.99 (0.98-1.00) | <0.001 | 1.00 (0.99-1.00) | 0.303 | 0.281 |
| BMI, kg/m^2^ | 1.02 (0.99-1.06) | 0.206 | 1.01 (0.96-1.07) | 0.605 | 0.820 |
| eGFR, mL/min/1.73 m^2^ | 0.97 (0.96-0.97) | <0.001 | 0.98 (0.97-0.98) | <0.001 | 0.077 |
| WBC, ×10^9^/L | 0.97 (0.92-1.03) | 0.309 | 1.04 (0.96-1.13) | 0.315 | 0.169 |
| Monocyte, ×10^9^/L | 2.18 (1.01-4.70) | 0.047 | 1.87 (0.54-6.52) | 0.325 | 0.533 |
| Calcium, mmol/L | 2.65 (1.12-6.26) | 0.027 | 1.15 (0.30-4.43) | 0.835 | 0.681 |
| Potassium, mmol/L | 1.20 (0.94-1.52) | 0.135 | 1.50 (1.05-2.14) | 0.027 | 0.984 |
| BNP, pg/mL | 1.00 (1.00-1.00) | <0.001 | 1.00 (1.00-1.00) | 0.021 | 0.229 |
| Albumin, g/L | 1.04 (1.01-1.07) | 0.022 | 1.06 (1.01-1.12) | 0.019 | 0.442 |
| Globulin, g/L | 1.03 (1.01-1.05) | 0.011 | 1.01 (0.97-1.05) | 0.666 | 0.496 |
| Triglyceride, mmol/L | 1.08 (0.87-1.35) | 0.486 | 1.18 (0.83-1.67) | 0.366 | 0.475 |
| HDL-C, mmol/L | 0.43 (0.28-0.65) | <0.001 | 0.30 (0.15-0.61) | <0.001 | 0.942 |
| Statin, n (%) |  |  |  |  | 0.936 |
| No | 1.00 |  | 1.00 |  |  |
| Yes | 0.81 (0.61-1.06) | 0.119 | 0.74 (0.48-1.14) | 0.173 |  |
| ACEI/ARB, n (%) |  |  |  |  | 0.602 |
| No | 1.00 |  | 1.00 |  |  |
| Yes | 0.84 (0.64-1.12) | 0.235 | 0.72 (0.46-1.11) | 0.141 |  |
| Diuretic, n (%) |  |  |  |  | 0.704 |
| No | 1.00 |  | 1.00 |  |  |
| Yes | 1.36 (0.56-3.28) | 0.495 | 2.48 (0.38-16.08) | 0.341 |  |
| Beta-blocker, n (%) |  |  |  |  | 0.838 |
| No | 1.00 |  | 1.00 |  |  |
| Yes | 1.13 (0.85-1.51) | 0.388 | 1.14 (0.72-1.78) | 0.581 |  |

Abbreviations: NYHA, New York Heart Association; SBP, systolic blood pressure; BMI, body mass index; eGFR, estimated glomerular filtration rate; WBC, white blood cell; BNP, B-type natriuretic peptide; HDL-C, high-density lipoprotein cholesterol; ACEI/ARB, angiotensin-converting enzyme inhibitor/angiotensin receptor blocker.
